# Cariprazine in multiple-episode dual schizophrenia: post-hoc analysis of an observational study

**DOI:** 10.1192/j.eurpsy.2025.625

**Published:** 2025-08-26

**Authors:** N. Szerman, Z. B. Dombi, P. Vega, C. Roncero, L. Peris, L. Grau-López, I. Basurte-Villamor

**Affiliations:** 1 Gregorio Marañón University Hospital, Madrid, Spain; 2 Gedeon Richter Plc., Budapest, Hungary; 3 Francisco de Vitoria University, Madrid; 4 University of Salamanca Healthcare Complex, Salamaca, Spain; 5 Centre Neuchâtelois De Psychiatrie, Marin-Epagnier, Switzerland; 6 University Hospital Vall d’Hebron, Barcelona, Spain

## Abstract

**Introduction:**

Schizophrenia frequently coexists with substance use disorders, especially cannabis use disorder (CUD). Importantly, cannabis use is known to further exacerbate symptoms, leading to more frequent and severe psychotic episodes, longer hospital stays and poorer overall treatment outcomes. The lifetime risk of relapse is around 60%. Therefore, the treatment of patients with multiple episodes is even more challenging

**Objectives:**

To evaluate the effectiveness of cariprazine in schizophrenia patients with multiple episodes and comorbid cannabis use disorder.

**Methods:**

This was a 6-month, multi-centre, observational study conducted at six institutions in Spain. The study included adult outpatients aged 18 to 65 years, diagnosed with schizophrenia and cannabis use disorder according to the Diagnostic and Statistical Manual of Mental Disorders (DSM-5) criteria, who were receiving cariprazine treatment based on medical judgment. Exclusion criteria included pregnant or breastfeeding women and patients with co-existing medical conditions that could potentially skew the study results.

The study evaluated changes in schizophrenia symptoms using the Positive and Negative Syndrome Scale (PANSS) and the Clinical Global Impression-Schizophrenia (CGI-SCH), as well as changes in CUD symptoms based on the Cannabis Abuse Screening Test (CAST) and the Severity of Dependence Scale (SDS). This post-hoc analysis focused on patients with multiple episodes. Patient characteristics were summarized using descriptive statistics. Least squares (LS) means were calculated for the change from treatment start to treatment end for PANSS, CGI-SCH, CAST, SDS using a mixed model for repeated measures. All analyses were conducted using SAS.

**Results:**

From the cohort, 38 (65%) patients had multiple episodes. The mean age of these patients was 36.5 and 65.8% of them was male. Most patients received 4.5 mg/day cariprazine at baseline (60.5%). Half of the patients also took concomitant antidepressants and/or antipsychotics. Throughout the 6-month observational period significant improvement was detected in both schizophrenia (LS mean change in PANSS Total score: -46.2, p<0.001, CGI-SCH Total score: -8.0, p<0.001) and CUD symptomatology (LS mean change in CAST Total score: -6.6, p<0.001, SDS Total score: -8.0, p<0.001).

**Conclusions:**

Cariprazine seem to be an effective treatment option for schizophrenia patients with multiple episodes and comorbid CUD.

**Disclosure of Interest:**

N. Szerman : None Declared, Z. Dombi Employee of: I am an employee of Gedeon Richter Plc., originator of cariprazine., P. Vega: None Declared, C. Roncero: None Declared, L. Peris: None Declared, L. Grau-López: None Declared, I. Basurte-Villamor: None Declared

